# P05.04. Developing a dialogue between refugee patients and healthcare providers about traditional medicine use

**DOI:** 10.1186/1472-6882-12-S1-P364

**Published:** 2012-06-12

**Authors:** K Fenn, S Crosby, A Filippelli, M Grodin, P Gardiner

**Affiliations:** 1 Boston University School of Medicine, Boston, USA

## Purpose

Traditional medicine use is common and diverse among patients in the United States. Many do not tell healthcare providers about their traditional medicine use or remedies nor do healthcare providers typically have the time to ask. This creates a barrier to the care received because the patient and healthcare provider do not communicate fully about treatment options. Our goal with this study was to increase communication about traditional and integrative medicine by putting together a survey following the analysis of these ethnographic interviews.

## Methods

Working within the Boston Center for Refugee Health and Human Rights at Boston Medical Center, we interviewed refugee and asylum seeking patients and their healthcare providers about traditional medicine use. This included a demographic survey and qualitative, open-ended interviews.

## Results

We formally interviewed 27 refugee and asylum-seeking patients and spoke with several healthcare providers throughout the study. The majority of interviewees were female (n=22) and from Africa (n=19), reflecting the demographic of patients throughout the clinic. Eighteen patients we interviewed reported using herbal remedies at some point in their lives, more than half (n=13) in the United States. Participants were much more open to discussing herbal medicine and religious healing than other types (e.g. ancestor worship).

## Conclusion

Through this survey, we hope to increase health care practitioners’ awareness of these issues and help them effectively navigate this conversation topic. Demonstrated understanding of their patients’ views of disease and medicine will potentially help the patients feel more comfortable in the clinic. In addition, it will enable both sides to be as open as possible with one another about treatment pathways.

